# Searching for New Z-DNA/Z-RNA Binding Proteins Based on Structural Similarity to Experimentally Validated Zα Domain

**DOI:** 10.3390/ijms23020768

**Published:** 2022-01-11

**Authors:** Martin Bartas, Kristyna Slychko, Václav Brázda, Jiří Červeň, Christopher A. Beaudoin, Tom L. Blundell, Petr Pečinka

**Affiliations:** 1 Department of Biology and Ecology, Faculty of Science, University of Ostrava, 710 00 Ostrava, Czech Republic; martin.bartas@osu.cz (M.B.); P21097@student.osu.cz (K.S.); jiri.cerven@osu.cz (J.Č.); 2 Department of Biophysical Chemistry and Molecular Oncology, Institute of Biophysics of the Czech Academy of Sciences, 612 65 Brno, Czech Republic; vaclav@ibp.cz; 3 Department of Biochemistry, Sanger Building, University of Cambridge, Tennis Court Rd., Cambridge CB2 1GA, UK; cab233@cam.ac.uk (C.A.B.); tlb20@cam.ac.uk (T.L.B.)

**Keywords:** Z-DNA, Z-RNA, Zα domain, protein binding, bioinformatics

## Abstract

Z-DNA and Z-RNA are functionally important left-handed structures of nucleic acids, which play a significant role in several molecular and biological processes including DNA replication, gene expression regulation and viral nucleic acid sensing. Most proteins that have been proven to interact with Z-DNA/Z-RNA contain the so-called Zα domain, which is structurally well conserved. To date, only eight proteins with Zα domain have been described within a few organisms (including human, mouse, *Danio rerio*, *Trypanosoma brucei* and some viruses). Therefore, this paper aimed to search for new Z-DNA/Z-RNA binding proteins in the complete PDB structures database and from the AlphaFold2 protein models. A structure-based similarity search found 14 proteins with highly similar Zα domain structure in experimentally-defined proteins and 185 proteins with a putative Zα domain using the AlphaFold2 models. Structure-based alignment and molecular docking confirmed high functional conservation of amino acids involved in Z-DNA/Z-RNA, suggesting that Z-DNA/Z-RNA recognition may play an important role in a variety of cellular processes.

## 1. Introduction

**Figure 1:**
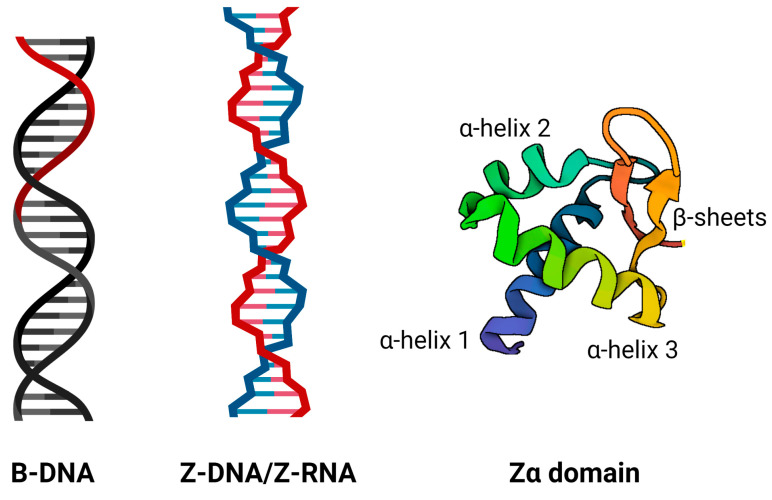
Schematic diagram of classical right-handed B-DNA, left-handed Z-DNA/Z-RNA, and Zα domain consisting of three α-helices and two β-strands. This domain is known to specifically interact with left-handed nucleic acids, mainly through its α-helix 3 and some amino acid residues of beta-strands.

Local DNA structures, also called ‘non-B’ DNA structures, have been recognised as important regulators of many fundamental regulatory processes, including replication [1], transcription [2], translation [3], epigenetics [4], DNA damage repair [5,6,7], genome evolution and rearrangement [8]. Negative supercoiling of DNA and protein binding can increase the stability of local DNA conformation and/or induce conformational changes that give rise to various alternative DNA structures, the best-described being cruciforms [7], Z-DNA/Z-RNA [9,10], triplexes [11] and quadruplexes [12]. Recently, a large number of proteins that recognise especially G-quadruplexes [13] and cruciforms [7,14] were characterised. Surprisingly, only a few Z-DNA/Z-RNA binding proteins have been characterised to date [15,16,17,18,19,20,21,22,23]. Z-DNA is a left-handed form of deoxyribonucleic acid, and its name was derived from the typical ‘zig-zag’ pattern (Figure 1). This DNA structure was first proposed by Robert Wells and his colleagues in 1970, during their physical and enzymatic studies on d(I–C) polymers (consisting of altered inosine and cytosine units) [24]. The first structure of Z-DNA was subsequently solved by Andrew H. Wang et al. in 1979 using complementary hexamers of d(CG)_3_ [25]. The next development was the crystallographic structure of the so-called B-Z junction (DNA loci where right-handed B-DNA passes to a left-handed Z-DNA conformation, or vice versa) [26]. Many biochemical and biophysical in vitro experiments have been conducted to better characterise Z-DNA behaviour at close to physiological conditions [27,28] and also to better understand switching between B and Z-DNA [29,30]. Furthermore, several bioinformatic searches have been performed to predict Z-DNA-prone sequence motifs in the genomic DNA of some model organisms, including humans [31,32]. Z-DNA structures can be formed only in specific double-stranded sequences with alternating purine–pyrimidine tracks, which has been determined by crystallography in various nucleotide repeats, where specifically Z-DNA containing GC repeats have been shown to have increased stability [33]. These sequences with a high potential to form Z-DNA were observed in the genomes of organisms across all domains of life, and their particular importance has been shown: e.g., in transposable ALU elements [34], and gene promoters [35]. In 2009, the first human map of experimentally-obtained Z-DNA forming sites was released [36], followed by the ChIP-seq map in 2016, where they associated Z-DNA forming sites with actively transcribed regions in the human genome [37]. Since these discoveries, it is clear that Z-DNA structures arise under physiological conditions. However, compared to classical B-DNA conformations, Z-DNA structures are energetically unfavourable and, therefore, the structure formation requires energy (usually in the form of negative supercoiling), which results in less structural stability [38].

In addition to Z-DNA, there is an analogous structure called Z-RNA (i.e., double-stranded left-handed RNA) that was firstly described in detail in 1984 by Kathleen Hall et al. [39]. Using a combination of spectroscopic techniques, they found that poly(GC)·poly(GC) undergoes a transition from the classical A-form to a left-handed Z-form. Z-RNA has also been found in viral genomes, For example, the influenza virus has been shown to produce Z-RNA during replication, which can induce ZBP1-mediated necroptosis [40]. Additionally, SARS-CoV-2 has been reported to contain loci that theoretically form Z-RNAs (not published, analysed in house using the Non-B DB webserver [41]) [33,34,35,40,41].

It is assumed that Z-DNA/Z-RNA structures often need ‘special’ binding proteins for their stabilisation. Most known Z-DNA binding proteins bind to left-handed nucleic acids through the so-called Z-DNA binding domain Zα (Figure 1). One of the first discovered human Z-DNA binding proteins was double-stranded RNA adenosine deaminase (now designated as ADAR1) in 1995 by Herbert et al. [42]. The Zα domain was also discovered in DAI, PKZ, E3L, and ORF112 proteins [21], and a recent study found that this domain is present in RBP7910 protein [43]. The structure of the Zα domain has a specific β-sheet-helix-turn-helix motif (βHTH), which is a subgroup of the winged HTH motif (wHTH). The Zα domain usually consists of three α-helices and sheets of two or three β-strands (αβααββ). The β-wing motif is formed by two antiparallel β-sheets composed of β2 and β3. The resulting β-wing and third α-helix play an important role in recognition and binding to Z-DNA [21,44].

During the past 40 years of research, only about ten Z-DNA (or Z-RNA) binding proteins have been identified in different organisms. All known Z-DNA/Z-RNA proteins that contain Zα domains have been demonstrated to be involved in the immune response (ADAR1, ZBP1, PKZ) [19,45,46,47,48] and/or virus-host interactions (E3L protein from *Vaccinia virus*, ORF112 protein from *Cyprinid herpesvirus* 3) [21,49,50,51]. Some studies have also shown that the binding of the Zα domain to Z-RNA is responsible for the localisation of Z-DNA/Z-RNA binding proteins into cytoplasmic stress granules [52,53,54]. One of the most well-characterised Z-DNA/Z-DNA binding proteins, ADAR 1, is, in fact, a moonlighting protein [55], and its Z-DNA/Z-RNA binding function was discovered [56] after it was originally described as an adenosine deaminase [57]. This led us to the hypothesis that some functionally characterised proteins may still possess an unidentified Z-DNA/Z-RNA binding function. Therefore, this paper aims to identify new Z-DNA/RNA binding proteins based on structural similarity to an experimentally well-defined Zα domain.

## 2. Results and Discussion

### 2.1. Prediction of New Z-DNA/Z-RNA Binding Proteins Based on Structural Similarity to the Experimentally Validated Zα Domain

At the beginning of our study, we made a list of experimentally solved Zα (and Zβ) domain structures (Table 1). After careful consideration (based mainly on the atomic-resolution and selection of a well-characterised human protein), we chose the crystal structure of the Zα domain from the human protein ADAR1 in complex with non-CG-repeat Z-DNA, obtained by Sung Chul Ha et al. in 2009 at a resolution of 2.20 Å [58]. Using this experimental Zα domain structure (PDB: 3f21, chain A), we carried out structural similarity searches using the PDBeFold web server (https://www.ebi.ac.uk/msd-srv/ssm/, (accessed on 10 September 2021)) and RUPEE web server (https://ayoubresearch.com/, (accessed on 21 October 2021)). The PDBeFold algorithm allows examination of a given protein structure for similarity with the whole PDB archive containing nearly 200k of experimentally solved protein structures from a variety of model and nonmodel organisms, whereas RUPEE allows the querying of protein structures predicted by AlphaFold2 [59].

In Table 2, all non-redundant hits with a Q-score higher than a predefined threshold are shown. The Q-score represents the quality function of the Cα alignment, maximised by the secondary structure matching (SSM) alignment algorithm [64]. The Q-score is reported in an interval from 0 to 1, where the Q-score reaches 1 in the case of identical structures and decreases with an increasing RMSD or a smaller alignment length. A Q-score of 0 indicates completely dissimilar structures. A Q-score higher than 0.1 can indicate some possibly significant level of structural similarity. Nonetheless, in this research, we set a more stringent Q-score threshold of 0.55. This value seemed to be meaningful as there were known structures of Z-DNA/Z-RNA binding proteins that scored below the newly reported domains (i.e., structures where the Z-DNA/Z-RNA binding function has not been described so far).

**Figure 2:**
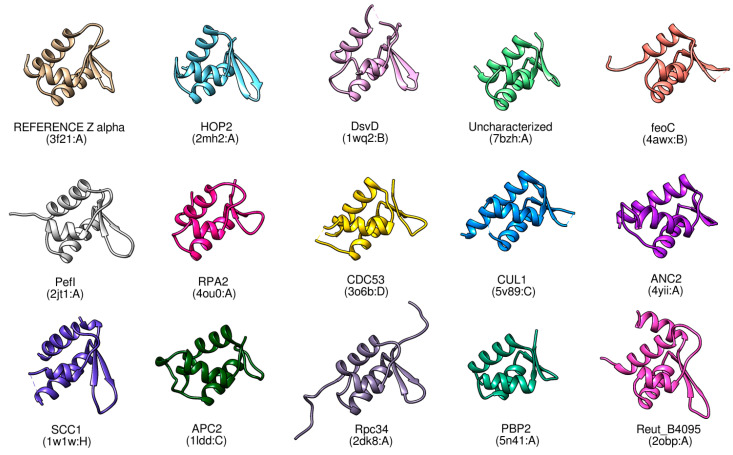
Comparison of the reference Zα domain (PDB: 3f21) (upper left corner) with the experimentally solved proteins (or their corresponding domains) having significant structural similarity (structures are ordered according to their similarity score to the reference structure (HOP2 best, DsvD second best, etc.).

The resulting hits from Table 2 are visualised in Figure 2, together with the “reference” structure of a Zα domain (PDB: 3f21), which was used as the query protein for the structural similarity searching. All 14 proteins show noticeable structural similarity to the functional Zα domain, as each of these structures contains three alpha-helices and two antiparallel beta-strands, in order, typical for the Zα domain.

The best new possible Z-DNA/Z-RNA binding protein found (based on the highest Q-score of its Zα domain), homologous-pairing protein 2 (HOP2), is widely conserved across the whole Eukarya domain. HOP2 proteins play an important role in meiotic recombination, particularly that of stimulating DMC1-mediated strand exchange that is necessary for homologous chromosome pairing during meiosis [81]. HOP2 forms a heterodimeric complex together with Meiotic nuclear division protein 1 homolog (MND1), and this HOP2/MND1 complex also promotes DMC1 mediated D-loop formation from double-strand DNA. Interestingly, a short 3bp deletion in the gene encoding HOP2 protein (leading to a deletion of a glutamic acid residue in the highly conserved C-terminal acidic domain) in humans causes “XX female gonadal dysgenesis” (XX-GD), which is a rare genetic disorder characterised for example by primary amenorrhea, uterine hypoplasia, or hypergonadotropic hypogonadism [82]. Another four proteins share a Cullin domain, particularly CDC53, CUL1, ANC2, and APC2. Proteins CDC53 (from *Saccharomyces cerevisiae*) and CUL1 (from *Homo sapiens*) are very distant functional homologs, and the same for ANC2 (from *Homo sapiens*) and APC2 (from *Saccharomyces cerevisiae*). Regarding Cullin domains and related ubiquitination processes, there are interesting links to viral diseases, see e.g., Rudnicka et al. [83]. Considering the current SARS-CoV-2 pandemic, it would be interesting to validate the potential of the viral RNA to form Z-RNA structures during replication, as was described for the influenza virus (H1N1 strain Puerto Rico/8/1934) virus in 2020 [40]. In this article, Zhang et al. found that replicating influenza A virus produces Z-RNAs and these are sensed by host ZBP1 in the nucleus of the host cell. This process led to the activation of specific protein kinases, resulting in nuclear rupture and unwanted necroptosis. From our newly described Z-DNA/Z-RNA binding proteins, protein Rpc34, which is subunit 6 of human RNA polymerase III, seems to have a direct association with a viral infection. For example, identical twins having a mutation in *POLR3F* (gene encoding Rpc34) had different susceptibility to the varicella-zoster virus in the CNS and lungs – the patient with the POLR3F mutation exhibited impaired antiviral and inflammatory responses and increased viral replication [84].

**Figure 3:**
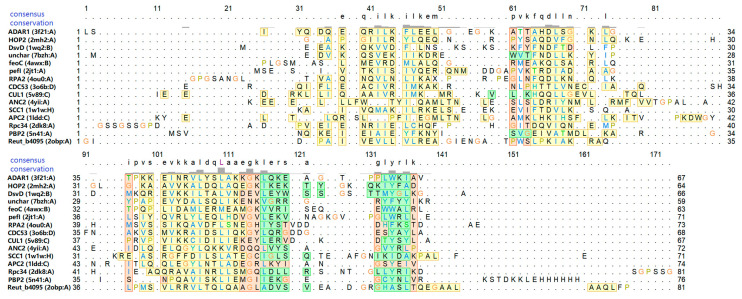
Sequence alignment is constructed from the structural superposition of the Zα domain of human ADAR1 protein (PDB: 3f21) and the 14 possible Z-DNA/Z-RNA binding proteins. The default colour of fully populated columns is light red, in addition, helices are coloured in yellow and strands in green. Letter colours correspond to the ClustalX colouring scheme.

Figure 3 shows a sequence alignment derived from the structural superposition of the predicted Zα domains from the analysed proteins to the Zα domain of the human protein ADAR1. All three alpha-helices are structurally conserved in the 14 possible Z-DNA/Z-RNA binding proteins. Similarly, beta-sheets of two or three strands are mostly preserved, except for in protein APC2. Interestingly, some amino acids in the predicted Zα domains were found to be repeatedly enriched in the exact positions of alignment—mainly in alpha helix 3, which is believed to be critical for Z-DNA/Z-RNA binding [52,60,85].

Most of these 14 proteins identified (except for proteins CDC53 and CUL1, and proteins ANC2 and APC2) do not likely share a common evolutionary ancestor. Instead, the similar global fold of Zα ‘domain’ could be a result of convergent evolution [86,87] leading to preferential Z-DNA/Z-RNA structures binding. Currently known Z-DNA/Z-RNA binding proteins (ADAR, ZBP1, PKZ, E3L) are also not homologous, but rather analogous in their Z-DNA/Z-RNA binding function. This phenomenon is common in the case of other proteins which preferentially bind noncanonical forms of nucleic acids, such as G-quadruplex binding proteins [88] or cruciform binding proteins [89] (most of them don’t have a common ancestor, but are analogous in their preferential interaction with G-quadruplexes, cruciforms, or another nucleic acid structures). In addition, it was found that some of the three-dimensional protein structures are widely conserved in non-homologous or unrelated DNA-binding proteins [90]. Then, the question arises we to whether the Zα domain is correctly annotated as a protein family (pfam ID: PF02295) as protein families are usually defined as groups of evolutionarily (not necessary functionally) related proteins. According to information deposited in the Pfam database, the HMM profile of this protein family was defined using only 5 seeds (regions 135–201 and 295–359 of human protein ADAR, region 137–203 of ADAR protein from *Rattus norvegicus*, region 7–71 of protein E3L from *Vaccinia virus*, and region 1–64 of protein ORF020 dsRNA-binding PKR inhibitor from *Orf virus* (Q6TVV0_ORFSA). This selection is problematic, as 3 of the 5 seed regions come from human and rat protein ADAR. The average length of the Zα domain is then 64.20 aa, with only 32% alignment identity. Therefore, we are sceptical about the current definition of the Zα domain on the level of the primary amino acid sequence. Nonetheless, further demystifying this issue is one motivation behind the scope of this paper, so we will continue with using the term ‘Zα domain’, in the sensu lato meaning, as the protein domain which preferentially interacts with Z-DNA/Z-RNA.

As the AlphaFold2 database [59] has provided putative structural models for thousands of proteins in several model organisms that have not yet been experimentally resolved, we sought to better understand which of these proteins may be involved in Z-DNA/Z-RNA binding. The ADAR1 Zα domain (PDB: 3f21) was chosen as a query structure for structural similarity searches using the RUPEE web server, which allows for the structural comparison with all AlphaFold2 models. RUPEE uses the TM-score to rank and quantify the structural similarity between protein alignments. On a scale from 0 to 1, a TM-score of over 0.5 is predicted to imply a similar fold. In a similar manner to the high Q-score threshold value used with PDBeFold, a TM-score of over 0.6 was chosen as a basis for the selection of hits from the structural alignment screen with RUPEE [91]. Since many of the proteins in the AlphaFold2 database do not yet have functional annotations, structural comparisons may further delineate their roles in cell survival.

Using the ADAR1 Zα domain (PDB: 3f21) as the query protein for the RUPEE web server, a total of 308 proteins were returned. Subsequent manual inspection of the alignments was performed to ensure that the putative Zα domains were structurally accessible and consisted primarily of basic residues that may be important for DNA-binding. A total of 185 unique proteins were selected after inspection, among which 59 proteins currently do not have complete functional annotation. Taking into consideration the previously annotated proteins that were predicted to contain one or more Zα domains, most have been assigned as putative transcriptional regulators—which further supports their potential to bind Z-DNA/Z-RNA. The probable [Fe-S]-dependent transcriptional repressor from *Escherichia coli* detected using RUPEE reflects the identification of the feoC protein from *Klebsiella* pneumoniae, detected using PDBeFold, that has been assigned the same function, which further validates the use of both structural comparison tools. In addition to feoC, additional similar proteins to Rpc34 and SCC1 were found, particularly DNA-directed RNA polymerase III subunit RPC3 (RNA polymerase III subunit C3) from *Leishmania infantum* and Rad21_Rec8 domain-containing protein from *Glycine max*. Interestingly, the uncharacterised proteins predicted to contain Zα domains were primarily found in the *Drosophila melanogaster*, *Methanocaldococcus jannaschii*, *Staphylococcus aureus*, and *Mycobacterium tuberculosis* proteomes (covering all three domains of life—Bacteria, Archaea, and Eukarya) The presence of proteins likely interacting with Z-DNA/Z-RNA in all domains of life further highlights the widespread occurrence of Z-DNA/Z-RNA and biological significance of such nucleic acid structures. The most numerous groups were uncharacterised proteins (59), transcriptional factors (56), and proteins related to ribosome biogenesis (49)—for further details see Supplementary Material S1. Both transcriptional factors and ribosomal proteins identified are in direct contact with DNA or RNA respectively, therefore their putative Z-DNA/Z-RNA binding ability is supported. The relatively large number of detected proteins, especially previously uncharacterised proteins, suggests that Z-DNA/Z-RNA binding domains may be more common than previously assumed. Further structural investigations may reveal the ability or extent of these proteins to bind Z-DNA/Z-RNA. Nonetheless, as the reliability of AlphaFold2 structural predictions still have some shortcomings [92], we have further proceeded only with 14 possible Z-DNA/Z-RNA binding proteins obtained from PDBeFold searches (experimentally solved structures).

### 2.2. Domain Composition and Nuclear Localisation Signals within the Most Promising Z-DNA/Z-RNA Binding Proteins

**Figure 4:**
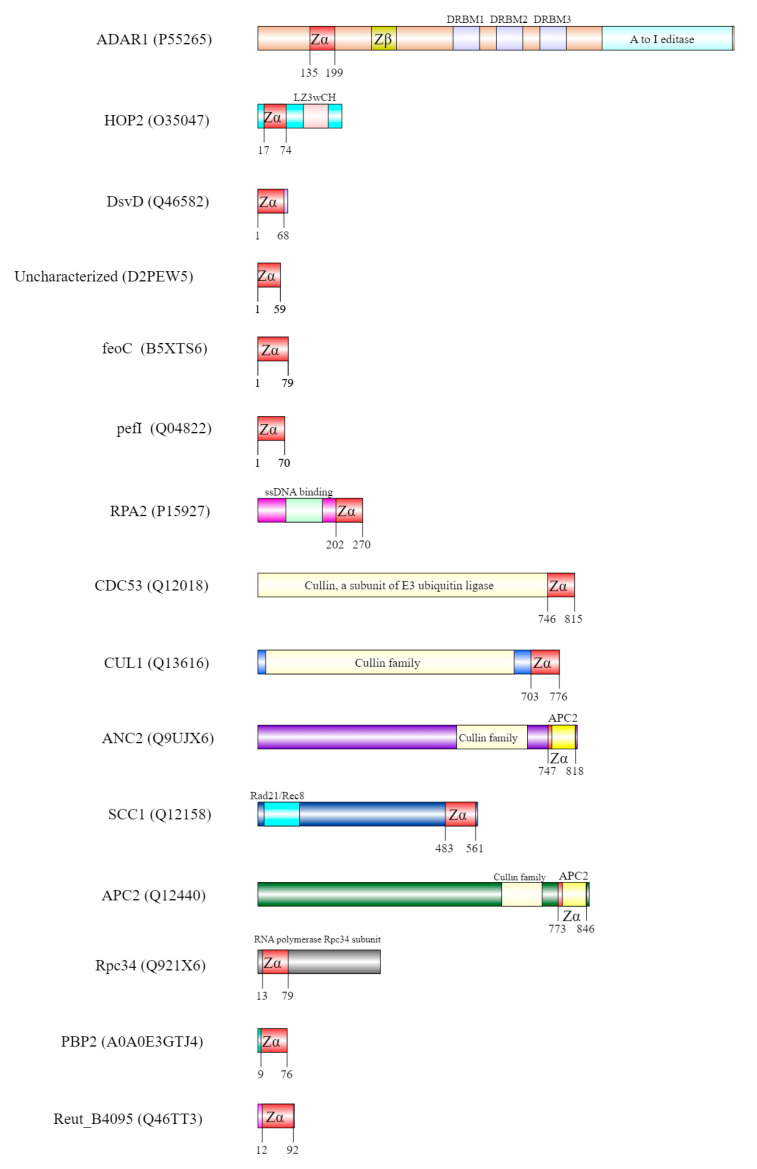
Position of Zα domain in ADAR1 and within 14 newly described possible Z-DNA/Z-RNA binding proteins. The exact position of Zα and its structural homologs is always highlighted in yellow.

Figure 4 shows the position of regions that are structurally similar to the Zα domain of ADAR1 and the 14 possible Z-DNA/RNA binding proteins inferred in the PDBeFold search (Table 2 and Figure 2). Interestingly, these regions are exclusively located in the N’ (HOP2, Rpc34) or C′ terminal ends (RPA2, CDC53, CUL1, ANC2, SCC1, APC2) of proteins longer than 100 aa. These data are in congruence with a previous observation by Chiang et al. [43], where they depicted the position of Zα domains in six proteins with known Z-DNA/RNA function (Zα domains were always located at the N terminal end of longer proteins). These results potentially highlight the need for maximal exposure of the Zα domain to be able to interact with this type of non-canonical nucleic acid structure. AlphaFold structures of predicted Z-DNA/Z-RNA binding proteins from *Homo sapiens* are enclosed in Supplementary Material S2, together with highlighted domains with structural similarity to Zα. In addition, in protein HOP2, there is an isoform lacking the N-terminal region (ΔN) spanning the Zα domain structural homolog. In the study conducted by Uanschou et al. they found that the N’ terminal domain of the protein HOP2 is crucial for its DNA-binding function in *Arabidopsis thaliana* [93]. Nevertheless, HOP2 protein seems to be highly conserved across Eukaryotic organisms (typical N-terminal wHTH was predicted also in the mouse, rat, human, *Saccharomyces cerevisiae* and *Dictyostelium discoideum* proteomes according to models obtained from AlphaFold2 database—https://alphafold.ebi.ac.uk/search/text/hop2, (accessed on 25 October 2021)) [59]. The above-mentioned ΔN isoform is also present in the human proteome according to UniProt Sequence annotation (Isoform 3: Q9P2W1-3, aa residues 1–125 are missing). Finally, there are also two previously known examples of human proteins ADAR1 and DAI, where, in both cases, ΔN isoforms exist (which result in missing Zα domain). Regarding protein ADAR1, its short isoform ADAR1p110 is constitutively expressed and located in the nucleus, whereas the long isoform ADAR1p150 is interferon-inducible and undergoes shuffling between the cytoplasm and nucleus [94,95]. Both of these isoforms share a Zβ domain (which may not have Z-DNA-binding ability [60] and its function is still unknown [96]), A-to-I deaminase domain, three double-stranded RNA-binding domains, but the long P150 isoform has an extra Z-DNA/RNA-binding domain at its N-terminus [97].

All eukaryotic proteins found have at least theoretical possibility to be localised both in the cytoplasm and cell nucleus, as was checked in a literature search and using nuclear localisation signal prediction within primary amino acid sequences of these proteins (cNLS Mapper webserver, accessed from http://nls-mapper.iab.keio.ac.jp/cgi-bin/NLS_Mapper_form.cgi, (accessed on 11 November 2021)) [98] (Supplementary Material S3). It is worth mentioning that the overall amino acid composition of these fourteen proteins identified shows similar significant enrichments (isoleucine, lysine, aspartic acid) and depletion (cysteine) as observed previously by us [99].

### 2.3. Representative Molecular Docking of RPA2 Region Structurally Similar to Zα Domain and Z-DNA/Z-RNA

**Figure 5:**
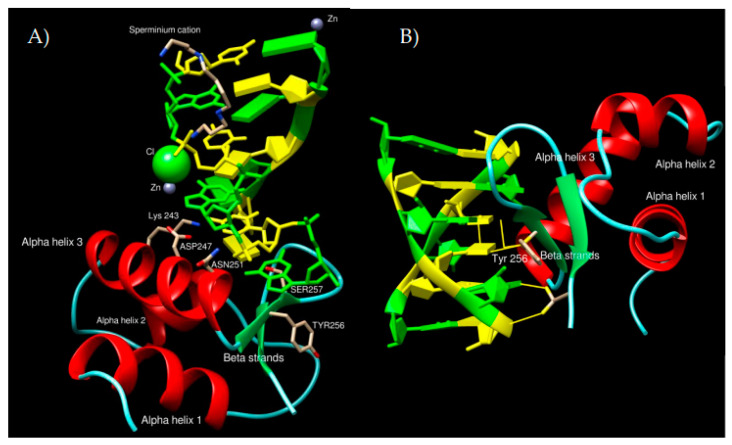
Representative molecular docking of human RPA2 Zα structural homolog to Z-DNA (**A**) and Z-RNA (**B**). Protein alpha helices are in red, beta-strands in green, coiled-coil regions in azure. Highlighting of Z-DNA/Z-RNA follows classic NDB colouring (guanines in green, cytosines in yellow).

We carried out representative molecular docking (using theHDOCK web server [100], further details in Materials and Methods section) of the human RPA2 putative Z-DNA/Z-RNA binding domain to Z-DNA (Figure 5A) and Z-RNA (Figure 5B). RPA2 was selected for its important molecular function in DNA replication and the cellular response to DNA damage. Results of this analysis revealed key amino acid residues involved in Z-DNA and/or Z-RNA binding. In both cases, tyrosine at position 256 (considering the whole RPA2 protein) was involved, suggesting its critical role in interaction with left-handed nucleic acids. In both cases, alpha-helix 3 and two subsequent beta-sheets seem to play pivotal roles in Z-DNA/Z-RNA recognition. These results are in congruence with previous experimental models of known Zα domains interacting with Z-DNA/Z-RNA, where the tyrosine, lysine, asparagine and serine amino acid residues played key roles in interaction [21,52,101,102]. The dockings of the remaining 13 possible Z-DNA/Z-RNA binding proteins are enclosed in Supplementary Material S4 (10 best docking poses for all protein/nucleic acid combinations). The inspection of the best docking poses revealed that it in general follows the rules described above. Carrying out a detailed molecular dynamic study would be beneficial in subsequent research to shed more light on the stability of these complexes.

### 2.4. Functional Enrichment and Interaction Network of Human Z-DNA/Z-RNA Binding Proteins

**Figure 6:**
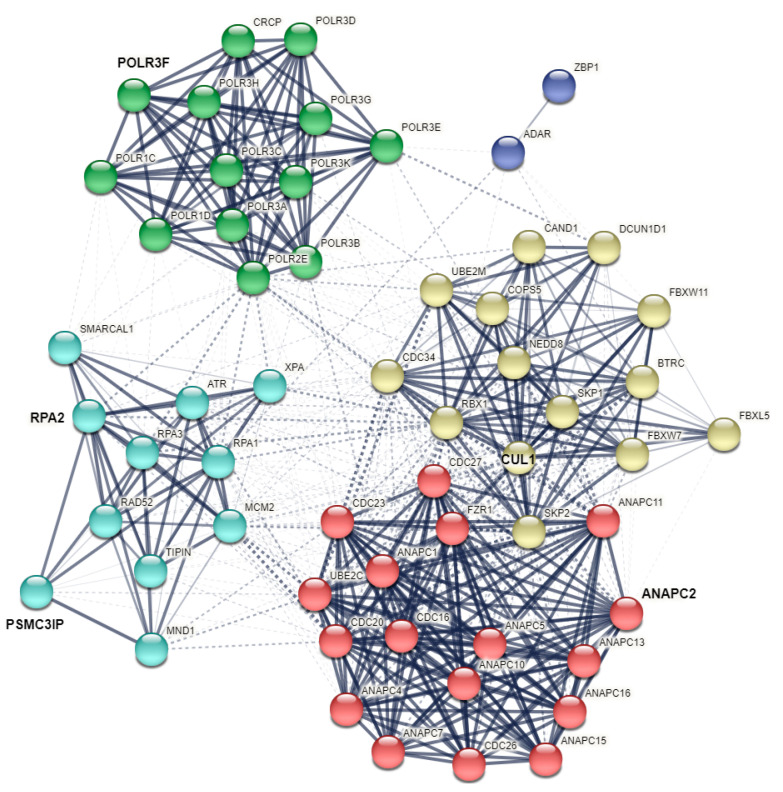
STRING interaction network of newly identified human possible Z-DNA/Z-RNA binding proteins (in bold and higher letter size), together with 2 previously known human Z-DNA/Z-RNA binding proteins (ZBP1 and ADAR), and also with 50 first shell interactors. Clustering was made using MCL inflation parameter (3), the resulting five clusters are highlighted in distinct colours. Line thickness indicates the strength of data support and edges between different clusters are dotted.

Finally, we aimed to better illustrate the possible functional interconnection between previously known human proteins ADAR and ZBP1, together with newly predicted human Z-DNA/Z-RNA binding proteins. We have constructed a STRING interaction network [103] made from two previously known human Z-DNA/Z-RNA binding proteins and five newly identified possible human Z-DNA/Z-RNA binding proteins containing structural similarity to the Zα domain. Additionally, the 50 closest interacting proteins were added via STRING (first shell of interactors) to better show possible pathways involving Z-DNA/Z-RNA binding and vice versa (Figure 6). This analysis has shown that newly identified possible Z-DNA/Z-RNA proteins (in humans) are quite distinct from two previously known human Z-DNA/Z-RNA interacting proteins ADAR and ZBP1 (blue cluster). Specifically, proteins RPA2 and HOP2 (syn. PSMC3IP) are both important members of the Meiotic Strand Invasion curated pathway [104] (azure cluster). POLR3F, the human homolog of mouse Rpc34, is interacting mainly with other subunits of RNA polymerase III complex, which is composed of 17 subunits and its structure was solved last year [105]. Interestingly, causative polymerase III mutations have been described in patients with hypersensitivity to viral infection [106,107]. The cluster containing human Cullin 1 protein (yellow) and a cluster containing ANAPC2 protein (red) are very tightly interconnected through functional interactions and involved in various cell cycle processes, including the proteasome-mediated ubiquitin-dependent protein catabolic process, the anaphase-promoting complex-dependent catabolic process, or activation of the innate immune response [108]. These results (Figure 6) reflect the current state of knowledge and do not consider the putative Z-DNA/Z-RNA binding function of proteins POLR3F, RPA2, HOP2/PSMC3IP, CUL1 and ANAPC2, which was first proposed in this manuscript. Once these proteins are validated as *bona fide* Z-DNA/Z-RNA binding in vitro (and their annotations are actualised within the STRING database), they will probably form a strong functional network by themselves (based on their Z-DNA/Z-RNA annotations).

## 3. Materials and Methods

### 3.1. Collection of Experimentally-Validated Z-DNA/RNA Binding Protein Structures

A systematic review of existing literature sources deposited in the Web of Science (https://clarivate.com/webofsciencegroup/solutions/web-of-science/, (accessed on 18 August 2021)), NCBI PubMed (https://pubmed.ncbi.nlm.nih.gov/, (accessed on 18 August 2021)), or Google Scholar (https://scholar.google.com/, (accessed on 18 August 2021)) databases was done to identify all up-to-date known Z-DNA/RNA binding proteins containing at least one Zα or Zβ domain. The resulting list of these proteins can be found in Table 1. Where available, the information about experimentally solved 3D structures was gathered as well.

### 3.2. Structure-Based Similarity Searches

Structure-based similarity searches were performed using the PDBeFold and RUPEE web servers [64], accessed from https://www.ebi.ac.uk/msd-srv/ssm/cgi-bin/ssmserver, (accessed on 10 September 2021), and from https://ayoubresearch.com/, (accessed on 21 October 2021). As a query, the experimentally-resolved structure of the Zα domain was used (PDB: 3f21, chain:A). PDBeFold was used to structurally compare the query Zα domain to all known experimentally-resolved structures in PDB, and RUPEE was used to query against all AlphaFold2 models. Parameters were left to be Default using PDBeFold, except for the “precision”, which was changed from “normal” to “high”. Three settings were used for the RUPEE search: “Full-Length” (finding exact length matches of the query protein in the database protein), “Contains” (finding query protein inside database protein), and “Contained-In” options (small protein motif detection in query protein). The hits resulting from the “Full-Length”, “Contained-In”, and “Contains” modes using RUPEE were combined to identify the total list of putative unique proteins.

### 3.3. Structure Visualisation and Contacts/Clashes Depicting

All protein structures were visualised and graphically pre-processed in a standalone version of the UCSF Chimera Tool [109]. Prediction of contact amino acid residues was carried out using the Chimera function “Find clashes/contacts” with the following parameters: “VDW overlap” ≥ 0.4 angstroms; “subtractions of 0.4 from overlap for potentially H-bonding pairs”; “Ignoring contacts of pairs 2 or fewer bonds apart”.

### 3.4. Structural Alignment Construction

Structural alignments of newly described Z-DNA/RNA binding proteins were done using Chimera structural analyses toolbox [110], particularly MatchMaker program was used with the following parameters: “Reference structure”: 3f21; “Structures to match”: 14 newly predicted proteins; “Chain pairing”: Best aligning pair of chains between reference and match structures; “Alignment algorithm”: Needleman-Wunsch; “Matrix”: BLOSUM-62; “Gap opening penalty”: 12; “Gap extension penalty”: 1; “Include secondary structure score”: 50%; “Compute secondary structure assignments“: yes; “Iterate by pruning long atom pairs until no pair exceeds”: 2.0 angstroms; “After superposition, compute structure-based multiple sequence alignment”: yes; “Create alignment from superposition”: choose all 15 protein structures; “Residue-residue distance cutoff”: 5.0 angstroms; “Residue aligned in column if within cutoff of”: at least one other; “Allow for circular permutation”: no; “Iterate superposition/alignment”: no.

### 3.5. Docking to Z-DNA/RNA

Docking of the putative RPA2 Zα domain (PDB: 4ou0:A) to Z-DNA (PDB: 4HIF) [111] and Z-RNA (PDB: 1T4X) [112] was done using HDOCK webserver (http://hdock.phys.hust.edu.cn/, (accessed on 30 December 2021)) [100] with default parameters. Protein structures were always submitted as a “receptor”, and Z-DNA structure as a “ligand”. The same procedure was repeated for the rest of the 14 possible Z-DNA/Z-RNA binding proteins. The resulting docking poses (best 10) are enclosed in Supplementary Material S4. The resulting models are sorted according to their HDOCK docking energy scores (“model 1” has the best energy score). Finally, the docking results were manually validated with respect to the existing literature, where main contact residues were determined (see Section 2.3 in Results and Discussion section).

### 3.6. Functional Enrichment Analysis

Functional enrichment analysis of 14 predicted Z-DNA/RNA binding proteins was done as follows: at first, homologous proteins were found in *Homo sapiens*, where available, and structural conservation of desired “Zα-like” fold was visually checked using AlphaFold prediction [59]. Secondly, five human proteins with conserved “Zα-like” fold (identified in this study) were uploaded to STRING webserver together with previously known Z-DNA/RNA binding proteins (https://string-db.org/cgi/input?sessionId=bVBUeCTKWYuE&input_page_show_search=on, (accessed on 12 December 2021)) [103] and 50 closest interacting proteins were automatically added via STRING (first shell of interactors).

## 4. Conclusions

Our analysis detected the Zα domain structural homologs in fourteen proteins that have not yet been described as Z-DNA/Z-RNA recognising proteins. These suggest that Z-DNA/Z-RNA recognition is more common and important in living systems than previously thought. Functional pathways interactions of the newly characterised proteins with a Zα domain indicate their involvement in innate immunity and other important molecular and biological pathways. These results also highlight the utility of structure-based similarity searches to elucidate the structure-function relationship of uncharacterised proteins or protein domains. Further experimental validation is required to determine the extent to which these proteins may bind to Z-DNA/Z-RNA.

## Figures and Tables

**Table 1:** Known Z-DNA/RNA binding proteins containing experimentally solved Zα or Zβ domain(s) (PDB IDs are provided). UniProtKB IDs of all proteins are provided as well.

Protein Symbol/ID	Protein Name	Organism	Protein Length	Function	PDB ID	Method/ Resolution	Domain	Ref.
ADAR (P55265)	Double-stranded RNA-specific adenosine deaminase	*Homo sapiens*	1226	Hydrolytic deamination of adenosine to inosine in dsRNA (A-to-I RNA editing)	1XMK	XRC/0.97 Å	Zβ	[60]
					1QGP	NMR	Zα	[61]
					3F21	XRC/2.20 Å	Zα	[58]
					3F22	XRC/2.50 Å	Zα	
					3F23	XRC/2.70 Å	Zα	
					2GXB	XRC/2.25 Å	Zα	[16]
ZBP1 (Q9H171)	Z-DNA-binding protein 1	*Homo sapiens*	429	Innate sensor recognising viral Z-RNA	2L4M	NMR	Zβ	[62]
Zbp1/DAI	Z-DNA-binding protein 1	*Mus musculus*	411		1J75	XRC/1.85 Å	Zα	[18]
PKZ (Q5NE12)	Protein kinase-containing Z-DNA-binding domains	*Danio rerio*	511	Defence response to virus	4LB5	XRC/2.00 Å	Zα	[20]
					4LB6	XRC/1.80 Å		
ORF112 (A4FTK7)	Protein ORF112	*Cyprinid herpesvirus* 3	278	Double-stranded RNA adenosine deaminase activity; RNA binding	4WCG	XRC/1.50 Å	Zα	[21]
E3L (P21605)	Protein E3	*Vaccinia virus*	190	Double-stranded RNA adenosine deaminase activity; inhibition of multiple cellular antiviral responses activated by dsRNA	7C0I	XRC/2.40 Å	Zα	[63]
34L (Q9DHS8)	34L protein	*Yaba-like disease virus*	185	Same as E3L	1SFU	XRC/2.00 Å	Zα	[22]

**Table 2:** Predicted Z-DNA/RNA binding proteins based on structural similarity to the experimentally validated Zα domain (3f21). Proteins are sorted according to their decreasing similarity score (Q-score); HOP2 is the best hit. UniProtKB IDs of all proteins are provided as well.

Protein Symbol/ID	Protein Name	Organism	Domain	Protein Length	Cellular Localisation/Known Function
HOP2 (O35047)	Homologous-pairing protein 2 homolog	*Mus musculus*	Eukarya	217	Nucleus/DNA binding, meiotic recombination, double-strand break repair, positive regulation of transcription by RNA pol II [65,66]
DsvD (Q46582)	DsvD	*Desulfovibrio vulgaris*	Bacteria	78	Role in dissimilatory sulfite reduction, Possible Interaction with B- and Z-DNA by Its Winged-Helix Motif [67]
D2PEW5	Uncharacterised DNA binding protein	*Sulfolobus islandicus*	Archaea	59	DNA binding
feoC (B5XTS6)	Probable [Fe-S]-dependent transcriptional repressor	*Klebsiella pneumoniae*	Bacteria	79	DNA binding may function as a transcriptional regulator that controls feoABC expression [68]
pefI (Q04822)	FaeA-like protein	*Salmonella typhimurium*	Bacteria	70	Regulation of transcription [69]
RPA2 (P15927)	Replication protein A 32 kDa subunit	*Homo sapiens*	Eukarya	270	Nucleus/DNA binding, multifunctional protein (DNA repairs, DNA replication, telomere maintenance, preventing G-quadruplex formation) [70,71,72,73]
CDC53 (Q12018)	Cell division control protein 53	*Saccharomyces cerevisiae*	Eukarya	815	Nucleus & Cytoplasm/DNA replication origin binding, cell division, protein ubiquitination [74]
CUL1 (Q13616)	Cullin-1	*Homo sapiens*	Eukarya	776	Nucleus & Cytoplasm/Protein ubiquitination, cell division, transcription regulation [75]
ANC2 (Q9UJX6)	Anaphase-promoting complex subunit 2	*Homo sapiens*	Eukarya	822	Nucleus & Cytoplasm/Component of the anaphase promoting complex/cyclosome (APC/C) [76]
SCC1 (Q12158)	Sister chromatid cohesion protein 1	*Saccharomyces cerevisiae*	Eukarya	566	Nucleus/Mitotic sister chromatid cohesion, double-strand break repair [77]
APC2 (Q12440)	Anaphase-promoting complex subunit 2	*Saccharomyces cerevisiae*	Eukarya	853	Nucleus & cytoplasm/Component of the anaphase promoting complex/cyclosome (APC/C) [78]
Rpc34 (Q921X6)	DNA-directed RNA polymerase III subunit RPC6	*Mus musculus*	Eukarya	316	Nucleus/Nuclear and cytosolic DNA sensor involved in innate immune response, defence response to the virus [79]
PBP2 (A0A0E3GTJ4)	Archaeal DNA polymerase holoenzyme (PBP2 subunit)	*Saccharolobus solfataricus*	Archaea	76	Enhances DNA synthesis [80]
Reut_B4095 (Q46TT3)	Putative DNA-binding protein	*Cupriavidus pinatubonensis*	Bacteria	95	DNA binding

## Data Availability

All data is contained within this article or Supplementary Material.

## Supplementary Materials

The following supporting information can be downloaded at: https://www.mdpi.com/article/10.3390/ijms23020768/s1, S1: RUPEE search through the whole AlphaFold database for proteins containing structural similarity to Zα domain; S2: AlphaFold structures of predicted human Z-DNA/Z-RNA proteins with highlighted Zα domains; S3: Nuclear localisation signals in newly described Z-DNA/Z-RNA binding proteins; S4: Top 10 docking poses for all possible Z-DNA/Z-RNA proteins and selected Z-DNA (PDB: 4HIF) and Z-RNA (PDB: 4HIF) structures.
