# Supplementary material for: Searching for New Z-DNA/Z-RNA Binding Proteins Based on Structural Similarity to Experimentally Validated Zα Domain
Source: Int J Mol Sci. 2022 Jan 11;23(2):768. doi: 10.3390/ijms23020768 (PMC8775963; doi:10.3390/ijms23020768)

Supplementary material 2 – AlphaFold structures of predicted Z-DNA/Z-RNA binding proteins from *Homo sapiens*

Note: Model confidence legends depicted below, position of Z-alpha domain structural similarity is always highlighted in red dashed circle

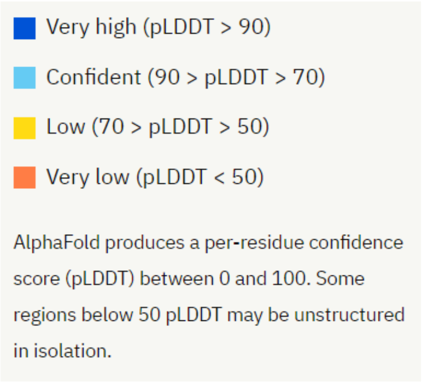

Accessed from: https://alphafold.ebi.ac.uk/

Citation:

Jumper, J et al. Highly accurate protein structure prediction with AlphaFold. Nature (2021).

Homologous-pairing protein 2 homolog Q9P2W1 (HOP2_HUMAN)

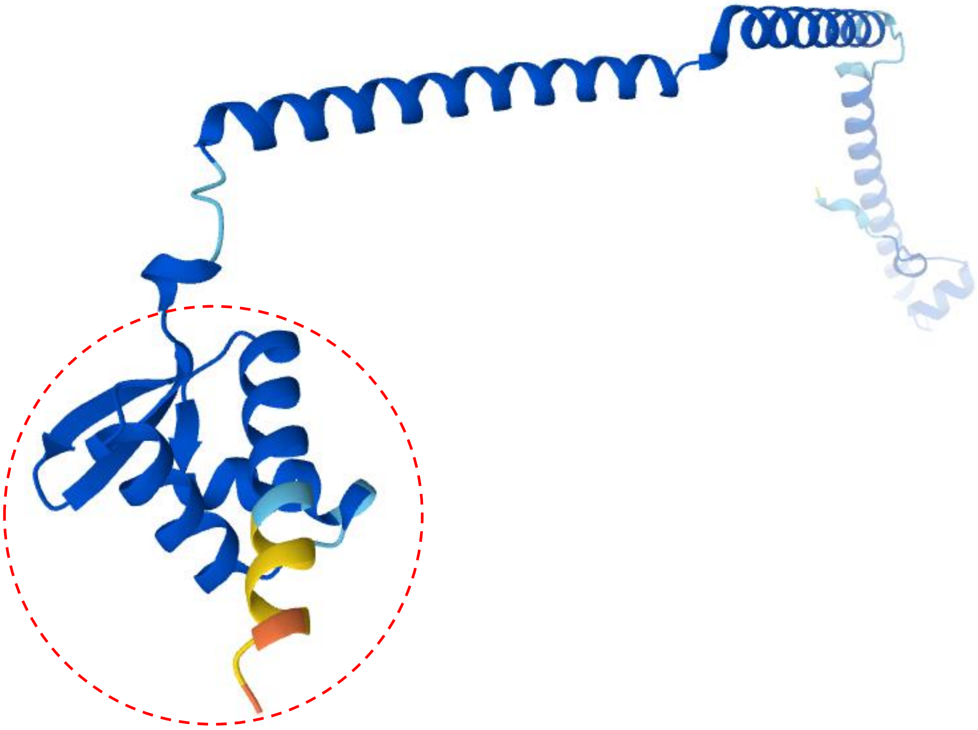

Replication protein A 32 kDa subunit P15927 (RFA2_HUMAN)

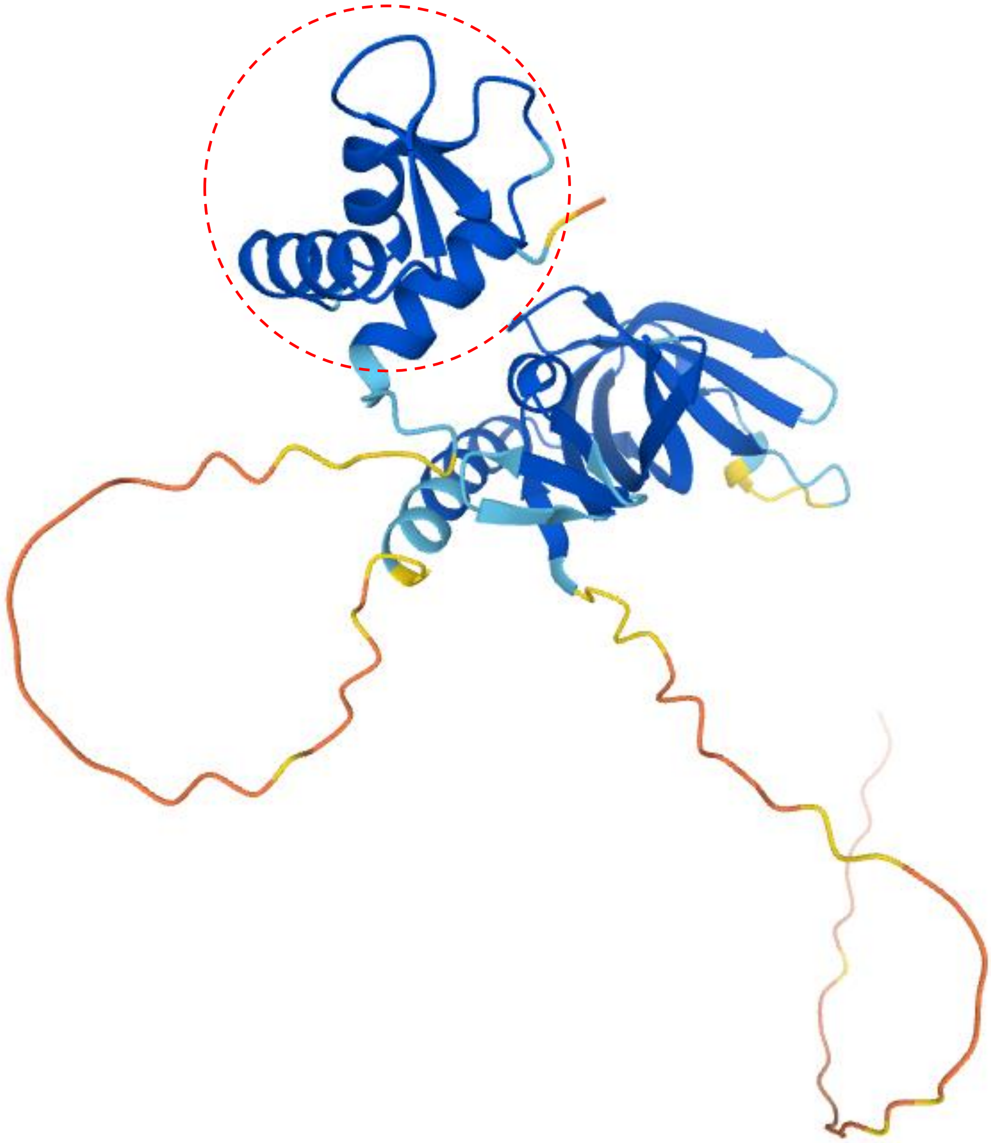

Cullin-1 Q13616 (CUL1_HUMAN)

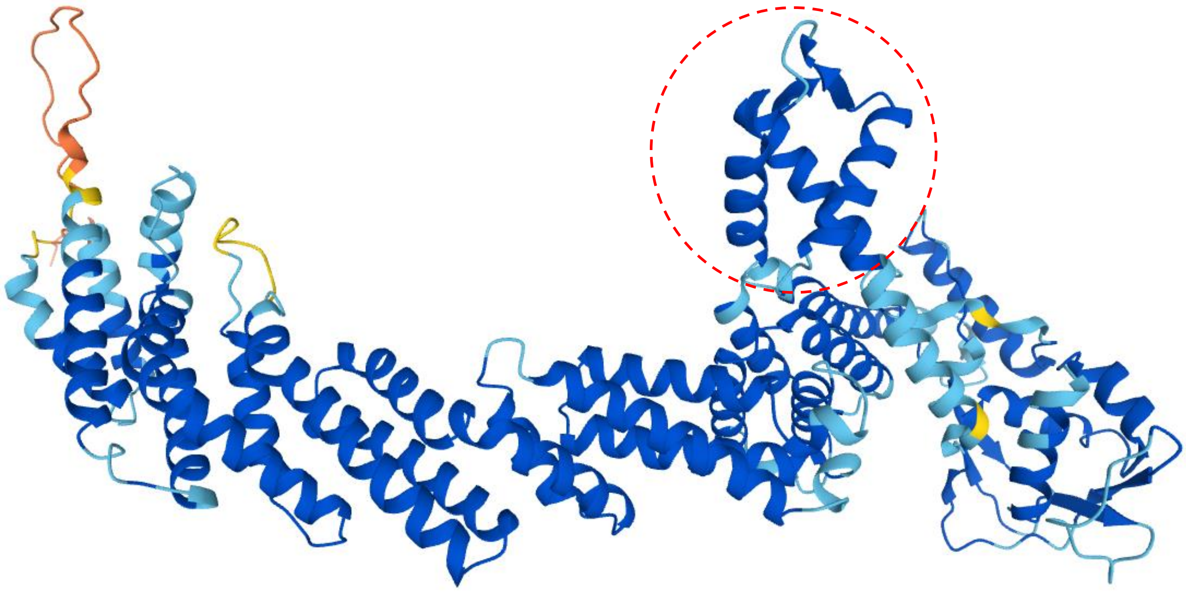

Anaphase-promoting complex subunit 2 Q9UJX6 (ANC2_HUMAN)

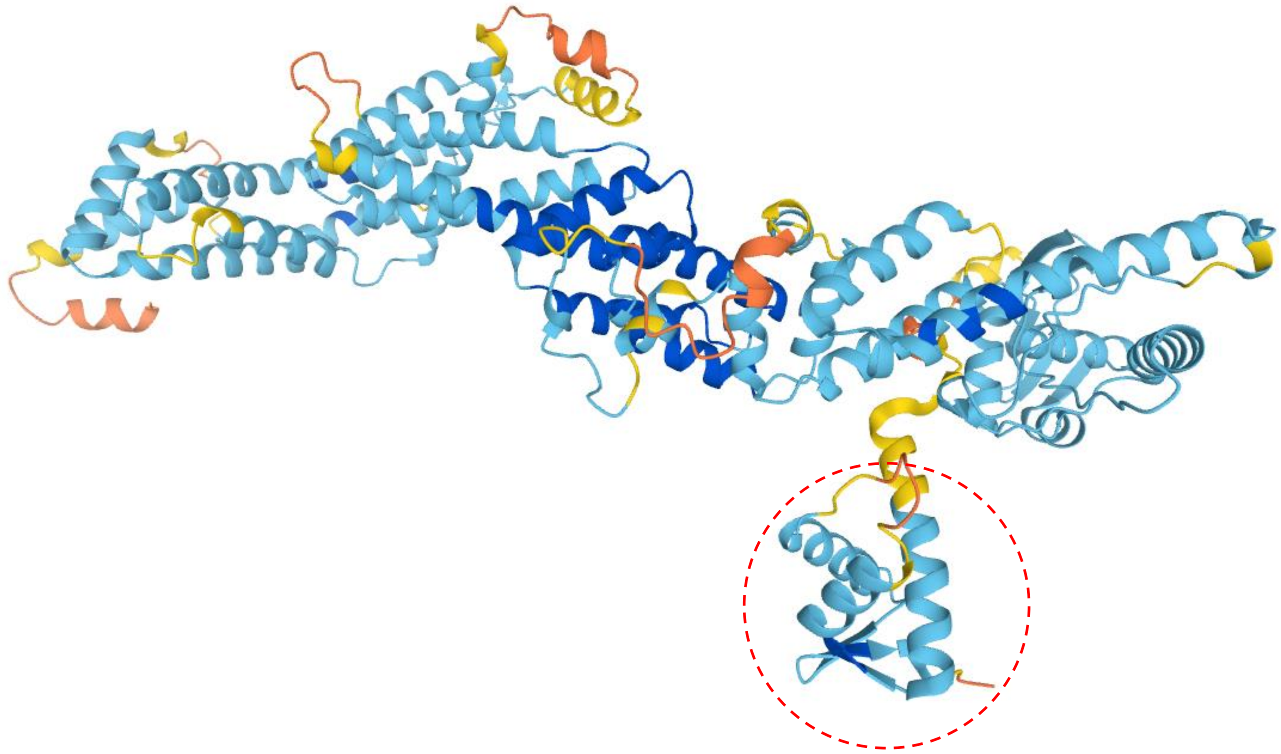

DNA-directed RNA polymerase III subunit RPC6 Q9H1D9 (RPC6_HUMAN)

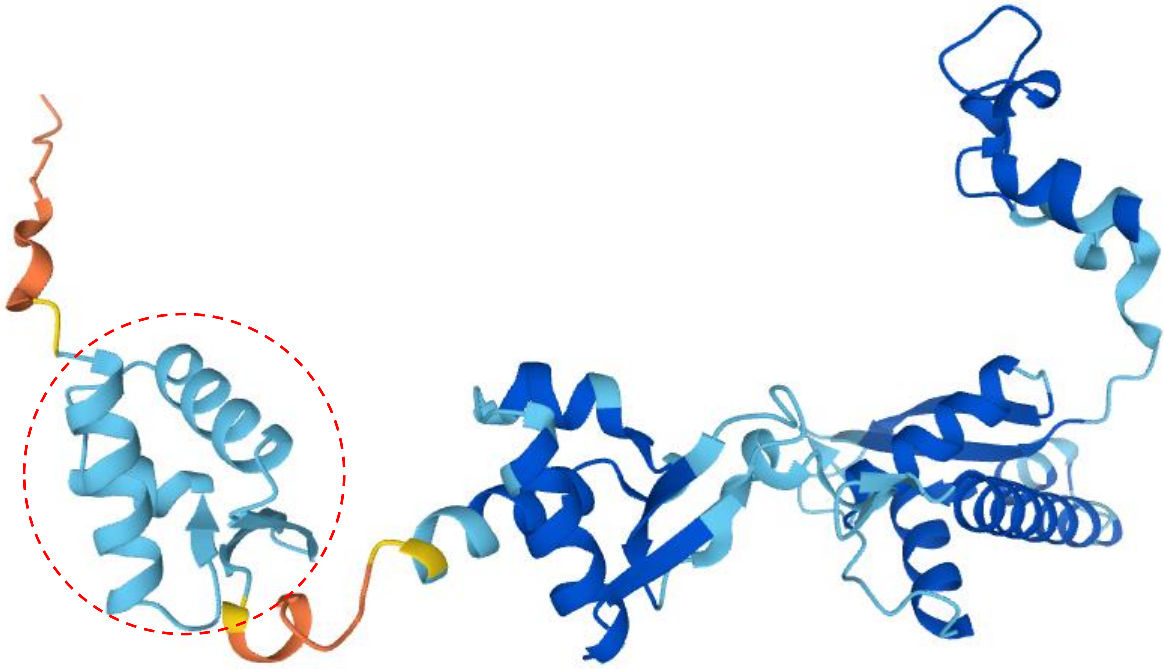

Supplement: Supplementary file 1 [file ijms-23-00768-s001.zip › Supplementary material 2.pdf]
