# Supplementary material for: Searching for New Z-DNA/Z-RNA Binding Proteins Based on Structural Similarity to Experimentally Validated Zα Domain
Source: Int J Mol Sci. 2022 Jan 11;23(2):768. doi: 10.3390/ijms23020768 (PMC8775963; doi:10.3390/ijms23020768)
Supplement: Supplementary file 1 [file ijms-23-00768-s001.zip › Supplementary material 3.pdf]

Supplementary material 3

Prediction of nuclear localization signals in putative Z-DNA/Z-RNA binding proteins, cNLS Mapper webserver was used (http://nls-mapper.iab.keio.ac.jp/cgi-bin/NLS_Mapper_form.cgi) with cut-off score equal to 3

>sp|O35047|HOP2_MOUSE Homologous-pairing protein 2 homolog OS=Mus musculus OX=10090 GN=Psmc3ip PE=1 SV=1

cNLS Mapper Result

Predicted NLSs in query sequence	
MSKSRAEAAAGAPGIILRYLQEQNRPYSAQDVFGNLQKEHGLGKAAVVKA	50
LDQLAQEGKIKEKTYGKQKIYFADQNQFDTVSDADLHGLDASIVALTAKV	100
QSLQQSCRHMEAELKELTSALTTPEMQKEIQELKKECAQYTERLKNIKAA	150
TNHVTPEEKEKVYRDRQKYCKEWRKRKRMTTELCDAILEGYPKSKKQFFE	200
EVGIETDEDHNVLLPDP	217

Predicted monopartite NLS

Pos.	Sequence	Score
172	EWRKRKRMTTELC	6
172	EWRKRKRMTT	8
174	RKRKRMTTE	3

Predicted bipartite NLS

Pos.	Sequence	Score
39	EHGLGKAAVVKALDQLAQEGKIKEKTYGKQKIY	3.1
142	ERLKNIKAATNHVTPEEKEKVYRDRQKYCKE	3.3
143	RLKNIKAATNHVTPEEKEKVYRDRQKYCKEW	3.4
164	RDRQKYCKEWRKRKRMTTELCDAILEGYPKSKK	4.9
166	RQKYCKEWRKRKRMTTELCDAILEGYPKSKKQFFEE	3.2
171	KEWRKRKRMTTELCDAILEGYPKSKK	7.6
171	KEWRKRKRMTTELCDAILEGYPKSKKQF	5.2
171	KEWRKRKRMTTELCDAILEGYPKSKKQFFE	8.1
171	KEWRKRKRMTTELCDAILEGYPKSKKQFFEE	6.9
171	KEWRKRKRMTTELCDAILEGYPKSKKQFFEE	4.8
172	EWRKRKRMTTELCDAILEGYPKSKK	6.4
172	EWRKRKRMTTELCDAILEGYPKSKKQF	3.7
172	EWRKRKRMTTELCDAILEGYPKSKKQFFE	8
172	EWRKRKRMTTELCDAILEGYPKSKKQFFEE	6.1

>sp|P15927|RFA2_HUMAN Replication protein A 32 kDa subunit OS=Homo sapiens OX=9606 GN=RPA2 PE=1 SV=1

cNLS Mapper Result

Predicted NLSs in query sequence
MWNSGFESYGSSSYGGAGGYTQSPGGFGSPAPSQAEKKSRARAQHIVPCT 50
ISQLLSATLVDEVFRIGNVEISQVTIVGIIRHAEKAPTNIVYKIDDMTAA 100
PMDVRQWVDTDDTSSENTVVPPETYVKVAGHLRSFQNKKSLVAFKIMPLE 150
DMNEFTTHILEVINAHMVLSKANSQPSAGRAPISNPGMSEAGNFGGNSFM 200
PANGLTVAQNQVLNLIKACPRPEGLNFQDLKNQLKHMSVSSIKQAVDFLS 250
NEGHIYSTVDDDHFKSTDAE 270

Predicted monopartite NLS		
Pos.	Sequence	Score

Predicted bipartite NLS		
Pos.	Sequence	Score
243	KQAVDFLSNEGHIYSTVDDDHFKSTDAE	3.5

>sp|Q12018|CDC53_YEAST Cell division control protein 53 OS=Saccharomyces cerevisiae (strain ATCC 204508 / S288c) OX=559292 GN=CDC53 PE=1 SV=1

Predicted NLSs in query sequence	
MSETLPRSDDLEATWNFIEPGINQILGNEKNQASTSKRVYKILSPTMYME	50
VYTAIYNYCVNKSRSSGHFSTDSRTGQSTILVGSEIYEKLKNYLKNYILN	100
FKQSNSETFLQFYVKRWKRFTIGAIFLNHAFDYMNRYWVQKERSDGKRHI	150
FDVNTLCLMTWKEVMFDPSKDVLINELLDQVTLGREGQIIQRSNISTAIK	200
SLVALGIDPQDLKKLNLNVYIQVFEKPFLKKTQEYYTQYTNDYLEKHSVT	250
EYIFEAHEIIKREEKAMTIYWDDHTKKPLSMALNKVLITDHIEKLENEFV	300
VLLDARDIEKITSLYALIRRDFTLIPRMASVFENYVKKTGENEISSLLAM	350
HKHNIMKNENANPKKLALMTAHSLSPKDYIKKLLEVHDIFSKIFNESFPD	400
DIPLAKALDNACGAFININEFALPAGSPKSATSKTSEMLAKYSDILLKKA	450
TKPEVASDMSDEDIITIFKYLTDKDAFETHYRRLFAKRLIHGTSTSAEDE	500
ENIIQRLQAANSMEYTGKITKMFQDIRLSKILEDDFAVALKNEPDYSKAK	550
YPDLQPFVLAENMWPFSYQEVEFKLPKELVPSHEKLKESYSQKHNGRILK	600
WLWPLCRGELKADIGKPGRMPFNFTVTLFQMAILLLYNDADVLTLENIQE	650
GTSLTIQHIAAAMVPFIKFKLIQQVPPGLDALVKPETQFKLSRPYKALKT	700
NINFASGVKNDILQSLSGGGHDNHGNKLGNKRLTEDERIEKELNTERQIF	750
LEACIVRIMKAKRNLPHTTLVNECIAQSHQRFNAKVSMVKRAIDSLIQKG	800
YLQRGDDGESYAYLA	815

Predicted monopartite NLS		
Pos.	Sequence	Score

Predicted bipartite NLS		
Pos.	Sequence	Score
36	SKRVYKILSPTMYMEVYTAIYNYCVNK	3.2
37	KRVYKILSPTMYMEVYTAIYNYCVNKSRSSG	3.2
88	EKLKNYLKNYILNFKQSNSETFLQFYVKRWK	3.2
91	KNYLKNYILNFKQSNSETFLQFYVKRWKRFTI	4
115	KRWKRFTIGAIFLNHAFDYMNRYWVQKERSD	3.3
426	GSPKSATSKTSEMLAKYSDILLKKATKPE	3.7
426	GSPKSATSKTSEMLAKYSDILLKKATKPEV	3
482	RRLFAKRLIHGTSTSAEDEENIIQRLQAAN	3
482	RRLFAKRLIHGTSTSAEDEENIIQRLQAANSME	3.2
665	PFIKFKLIQQVPPGLDALVKPETQFKLSRPY	6.4
666	FIKFKLIQQVPPGLDALVKPETQFKLSRPY	3.3
709	KNDILQSLSGGGHDNHGNKLGNKRLTED	3.8
757	RIMKAKRNLPHTTLVNECIAQSHQRFNAKVS	3.6
757	RIMKAKRNLPHTTLVNECIAQSHQRFNAKVS	3
757	RIMKAKRNLPHTTLVNECIAQSHQRFNAKVSM	5
757	RIMKAKRNLPHTTLVNECIAQSHQRFNAKVSMVK	3

>sp|Q13616|CUL1_HUMAN Cullin-1 OS=Homo sapiens OX=9606 GN=CUL1 PE=1 SV=2

Predicted NLSs in query sequence

```
MSSTRSQNPHGLKQIGLDQIWDDLRAGIQQVYTRQSMAKSRYMELYTHVY  50
NYCTSVHQSNQARGAGVPPSKSKKGQTPGGAQFVGLELYKRLKEFLKNYL  100
TNLLKDGEDLMDESVLKFYTQQWEDYRFSSKVLNGICAYLNRHWVRRECD  150
EGRKGIYEIYSLALVTWRDCLFRPLNKQVTNAVLKLIEKERNGETINTRL  200
ISGVVQSYVELGLNEDDAFAKGPTLTVYKESFESQFLADTERFYTRESTE  250
FLQQNPVTEYMKKAEARLLEEQRRVQVYLHESTQDELARKCEQVLIEKHL  300
EIFHTEFQNLLDADKNEDLGRMYNLVSRIQDGLGELKKLLETHIHNQGLA  350
AIEKCGEAALNDPKMYVQTVLDVHKKYNALVMSAFNNDAGFVAALDKACG  400
RFINNNAVTKMAQSSSKSPELLARYCDSLLKKSSKNPEEAELEDTLNQVM  450
VVFKYIEDKDVFQKFYAKMLAKRLVHQNSASDDAEASMISKLKQACGFEY  500
TSKLQRMFQDIGVSKDLNEQFKKHLTNSEPLDLDFSIQVLSSGSWPFQQS  550
CTFALPSELERSYQRFTAFYASRHSGRKLTWLYQLSKGELVTNCFKNRYT  600
LQASTFQMAILLQYNTEDAYTVQQLTDSTQIKMDILAQVLQILLKSKLLV  650
LEDENANVDEVELKPDTLIKLYLGYKNKKLRVNINVPMKTEQKQEQETTH  700
KNIEEDRKLLIQAAIVRIMKMRKVLKHQQLLGEVLTQLSSRFKPRVPVIK  750
KCIDILIEKEYLERVDGEKDTYSYLA                          776
```

Predicted monopartite NLS

Pos.	Sequence	Score
673	LGYKNKKLRVN	3

Predicted bipartite NLS

Pos.	Sequence	Score
63	RGAGVPPSKSKKGQTPGGAQFVGLELYKRLKEFL	4.2
90	KRLKEFLKNYLTNLLKDGEDLMDESVLKFYT	3.3
91	RLKEFLKNYLTNLLKDGEDLMDESVLKFYT	4.9
91	RLKEFLKNYLTNLLKDGEDLMDESVLKFYTQQWE	3
146	RRECDEGRKGIYEIYSLALVTWRDCLFRPLNKQVT	3
408	VTKMAQSSSKSPELLARYCDSLLKKSS	3.3
408	VTKMAQSSSKSPELLARYCDSLLKKSSKNP	6.7
424	RYCDSLLKKSSKNPEEAELEDTLNQVMVVFKYIEDK	4.5
464	KFYAKMLAKRLVHQNSASDDAEASMISKLK	3.1
468	KMLAKRLVHQNSASDDAEASMISKLKQA	3.5
468	KMLAKRLVHQNSASDDAEASMISKLKQACG	5.1
468	KMLAKRLVHQNSASDDAEASMISKLKQACGFE	3.7
489	ISKLKQACGFEYTSKLQRMFQDIGVSKDLNE	3.3
573	RHSGRKLTWLYQLSKGELVTNCFKNRYTLQAST	3
643	LLKSKLLVLEDENANVDEVELKPDTLIKLY	4.5
646	SKLLVLEDENANVDEVELKPDTLIKLYL	3.3
676	KNKKLRVNINVPMKTEQKQEQETTHKNIEED	4.1
697	ETTHKNIEEDRKLLIQAAIVRIMKMRKVLKH	6.3
717	RIMKMRKVLKHQQLLGEVLTQLSSRFKPRVP	4.4
717	RIMKMRKVLKHQQLLGEVLTQLSSRFKPRVPVIK	3.2
722	RKVLKHQQLLGEVLTQLSSRFKPRVPVIKKCI	4.6

>sp|Q9UJX6|ANC2_HUMAN Anaphase-promoting complex subunit 2 OS=Homo sapiens OX=9606 GN=ANAPC2 PE=1 SV=1

Predicted NLSs in query sequence	
MAAAVVVAEGDSDSRPGQELLVAWNTVSTGLVPPAALGLVSSRTSGAVPP	50
KEEELRAAVEVLRGHGLHSVLEEWFVEVLQNDLQANISPEFWNAISQCEN	100
SADEPQCLLLLLDAFGLLESRLDPYLRSLELLEKWTRLGLLMGTGAQGLR	150
EEVHTMLRGVLFFSTPRTFQEMIQRLYGCFLRVYMQSKRKGEGGTDPELE	200
GELDSRYARRRYYRLLQSPLCAGCSSDKQQCWCRQALEQFHQLSQVLHRL	250
SLLERVSAEAVTTTLHQVTRERMEDRCRGEYERSFLREFHKWIERVVGWL	300
GKVFLQDGPARPASPEAGNTLRRWRCHVQRFFYRIYASLRIEELFSIVRD	350
FPDSRPAIEDLKYCLERTDQRQQLLVSLKAALETRLLHPGVNTCDIITLY	400
ISAIKALRVLDPSMVILEVACEPIRRYLRTREDTVRQIVAGLTGDSDGTG	450
DLAVELSKTDPASLETGQDSEDDSGEPEDWVPDPVDADPGKSSSKRRSSD	500
IISLLVSIYGSKDLFINEYRSLLADRLLHQFSFSPEREIRNVELLKLRFG	550
EAPMHFCEVMLKDMADSRRINANIREEDEKRPAEEQPPFGVYAVILSSEF	600
WPPFKDEKLEVPEDIRAALEAYCKKYEQLKAMRTLSWKHTLGLVTMDVEL	650
ADRTLSVAVTPVQAVILLYFQDQASWTLEELSKAVKMPVALLRRRMSVWL	700
QQGVLREEPPGTFSVIEEERPQDRDNMVLIDSDDESDSGMASQADQKEEE	750
LLLFWTYIQAMLTNLESLSLDRIYNMLRMFVVTGPALAEIDLQELQGYLQ	800
KKVRDQQLVYSAGVYRLPKNCS	822

Predicted monopartite NLS		
Pos.	Sequence	Score

Predicted bipartite NLS		
Pos.	Sequence	Score
182	RVYMQSKRKGEGGTDPELEGELDSRYARR	5.1
182	RVYMQSKRKGEGGTDPELEGELDSRYARRRY	5.6
182	RVYMQSKRKGEGGTDPELEGELDSRYARRRYY	6.7
182	RVYMQSKRKGEGGTDPELEGELDSRYARRRYY	7.8
182	RVYMQSKRKGEGGTDPELEGELDSRYARRRYYR	6.7
182	RVYMQSKRKGEGGTDPELEGELDSRYARRRYY	3
182	RVYMQSKRKGEGGTDPELEGELDSRYARRRYYRL	3.2
182	RVYMQSKRKGEGGTDPELEGELDSRYARRRYYRLL	3.9
204	DSRYARRRYYRLLQSPLCAGCSSDKQQCWCR	3.8
206	RYARRRYYRLLQSPLCAGCSSDKQQCWCRQA	4
206	RYARRRYYRLLQSPLCAGCSSDKQQCWCRQALE	3.2
295	RVVGWLGKVFLQDGPARPASPEAGNTLRRWRCHV	3.7
401	ISAIKALRVLDPSMVILEVACEPIRRYLRT	3.5
401	ISAIKALRVLDPSMVILEVACEPIRRYLRTRE	3.4
491	KSSSKRRSSDIISLLVSIYGSKDLFINEYR	3.4
540	RNVELLKLRFGEAPMHFCEVMLKDMADSRRINA	4.2
604	FKDEKLEVPEDIRAALEAYCKKYEQLKAMRT	3.2

>sp|Q12158|SCC1_YEAST Sister chromatid cohesion protein 1 OS=Saccharomyces cerevisiae (strain ATCC 204508 / S288c) OX=559292 GN=MCD1 PE=1 SV=1

Predicted NLSs in query sequence
MVTENPQRLTVLRLATNKGPLAQIWLASNMSNIPRGSVIQTHIAESAKEI 50
AKASGCDDESGDNEYITLRTSGELLQGIVRVYSKQATFLLTDIKDTLTKI 100
SMLFKTSQKMTSTVNRLNTVTRVHQLMLEDAVTEREVLVTPGLEFLDDTT 150
IPVGLMAQENSMERKVQGAAPWDTSLEVGRRFSPDEDFEHNNLSSMNLDF 200
DIEEGPITSKSWEEGTRQSSRNFDTHENYIQDDDFPLDDAGTIGWDLGIT 250
EKNDQNNDDDDNSVEQGRRLGESIMSEEPTDFGFDLDIEKEAPAGNIDTI 300
TDAMTESQPKQTGTRRNSKLLNTKSIQIDEETENSESIASSNTYKEERSN 350
NLLTPQPTNFTTKRLWSEITESMSYLPDPILKNFLSYESLKKRKIHNGRE 400
GSIEEPELNVSLNLTDDVISNAGTNDNSFNELTDNMSDFVPIDAGLNEAP 450
FPEENIIDAKTRNEQTTIQTEKVRPTPGEVASKAIVQMAKILRKELSEEK 500
EVIFTDVLKSQANTEPENITKREASRGFFDILSLATEGCIGLSQTEAFGN 550
IKIDAKPALFERFINA 566

Predicted monopartite NLS		
Pos.	Sequence	Score
387	YESLKKRKIH	6.5

Predicted bipartite NLS		
Pos.	Sequence	Score
360	FTTKRLWSEITESMSYLPDPILKNFLSY	5.5
360	FTTKRLWSEITESMSYLPDPILKNFLSYE	3.8

>sp|Q12440|APC2_YEAST Anaphase-promoting complex subunit 2 OS=Saccharomyces cerevisiae (strain ATCC 204508 / S288c) OX=559292 GN=APC2 PE=1 SV=1

Predicted NLSs in query sequence

```
MSFQITPTRDLKVITDELQTLSSYIFHTNIVDDLNSLLTWMSPNDAKSNH  50
QLRPPSLRIKNIIKVLFPNNATTSPYSMINTSQANNSIVNEGNTNKELQL 100
QLFSTLKEFYIFQVRYHFFLHFNNINYLKDIQRWENYYEFPLRYVPIFDV 150
NVNDWALELNSLRHYLLNRNIKFKNNLRTRLDKLIMDDDFDLADNLIQWL 200
KSANGSLSSTELIVNALYSKINKFCEDNMSRVWNKRFMIMETFNKFINQY 250
WSQFSKLVGCPEDDHELTTTVFNCFESNFLRIRTNEIFDICVLAYPDSKV 300
TLLELRKIMKDFKDYTNIVTTFLSDFKKYILNPSVTTVDALLRYVKTIKA 350
FLVLDPTGRCLHSITTFVKPYFQERKHLVNVLLYAMLDLPEEELKEKINF 400
NVDMKALLSLVDTLHDSDINQDTNITKRDKNKKSPFLWNLKVKGKRELNK 450
DLPIRHAMLYEHILNYYIAWVPEPNDMIPGNIKSSYIKTNLFEVLLDLFE 500
SREFFISEFRNLLTDRLFTLKFYTLDEKWTRCLKLIREKIVKFTETSHSN 550
YITNGILGLLETTAPAADADQSNLNSIDVMLWDIKCSEELCRKMHEVAGL 600
DPIIFPKFISLLYWKYNCDTQGSNDLAFHLPIDLERELQKYSDIYSQLKP 650
GRKLQLCKDKGKVEIQLAFKDGRKLVLDVSLEQCSVINQFDSPNDEPICL 700
SLEQLSESLNIAPPRLTHLLDFWIQKGVLLKENGTYSVIEHSEMDFDQAQ 750
KTAPMEIENSNYELHNDSEIERKYELTLQRSLPFIEGMLTNLGAMKLHKI 800
HSFLKITVPKDWGYNRITLQQLEGYLNTLADEGRLKYIANGSYEIVKNGH 850
KNS                                                853
```

Predicted monopartite NLS

Pos.	Sequence	Score

Predicted bipartite NLS

Pos.	Sequence	Score
103	FSTLKEFYIFQVRYHFFLHFNNINYLKDIQRW	3.3
169	RNIKFKNNLRTRLDKLIMDDDFDLADNLIQWLK	3.7
174	KNNLRTRLDKLIMDDDFDLADNLIQWLKSAN	3.6
197	IQWLKSANGSLSSTELIVNALYSKINKFCED	3.2
220	KINKFCEDNMSRVWNKRFMIMETFNKFINQY	3
231	RVWNKRFMIMETFNKFINQYWSQFSKLVGCP	4.9
231	RVWNKRFMIMETFNKFINQYWSQFSKLVGCPE	3.4
304	ELRKIMKDFKDYTNIVTTFLSDFKKYILNP	3
325	DFKKYILNPSVTTVDALLRYVKTIKAFLV	3.7
372	FQERKHLVNVLLYAMLDLPEEELKEKINFN	3
372	FQERKHLVNVLLYAMLDLPEEELKEKINFNVD	4.1
428	RDKNKKSPFLWNLKVKGKRELNKDLPIRHAMLY	3
441	KVKGKRELNKDLPIRHAMLYEHILNYYIAWVP	4
486	YIKTNLFEVLLDLFESREFFISEFRNLLTD	3.4
516	RLFTLKFYTLDEKWTRCLKLIREKIVKFTETS	3.1
582	WDIKCSEELCRKMHEVAGLDPIIFPKFISL	3.3
588	EELCRKMHEVAGLDPIIFPKFISLLYWKYNCDT	3.2
649	KPGRKLQLCKDKGKVEIQLAFKDGRKLVLD	3.7
649	KPGRKLQLCKDKGKVEIQLAFKDGRKLVLDVS	3.1
747	DQAQKTAPMEIENSNYELHNDSEIERKYELT	3.8

>sp|Q921X6|RPC6_MOUSE DNA-directed RNA polymerase III subunit RPC6 OS=Mus musculus OX=10090 GN=Polr3f PE=1 SV=1

Predicted NLSs in query sequence
MAEVKVKVQPPDADPVEIENRIIELCHQFPHGITDQVIQNEMPHIEAQQR 50<br>AVAINRLLSMGQLDLLRSNTGLLYRIKDSQNAGKMKGSDNQEKLVYQIIE 100<br>DAGNKGIWSRDIRYKSNLPLTEINKILKNLESKKLIKAVKSVAASKKKVY 150<br>MLYNLQPDRSVTGGAWYSDQDFESEFVEVLNQQCFKFLQSKAETARESKQ 200<br>NPVIQRNSSFASSHEVWKYICELGISKVELSMEDIETILNTLIYDGKVEM 250<br>TIIAAKEGTVGSVDGHMKLYRAVNPILPPTGVVRAPCGLCPVFEDCHEGG 300<br>EISPSNCIYMTEWLEF 316

Predicted monopartite NLS		
**Pos.**	**Sequence**	**Score**

Predicted bipartite NLS		
**Pos.**	**Sequence**	**Score**
110	RDIRYKSNLPLTEINKILKNLESKKLIKA	4.1
110	RDIRYKSNLPLTEINKILKNLESKKLIKA	5.3
110	RDIRYKSNLPLTEINKILKNLESKKLIKAVK	6.9
122	EINKILKNLESKKLIKAVKSVAASKKKVYM	3.2
